# The Evaluation of Microcarcinoma in Differentiated Thyroid Cancers According to Old and New TNM Classification

**DOI:** 10.4274/MIRT.13

**Published:** 2011-12-01

**Authors:** Zekiye Hasbek, Bülent Turgut, Taner Erselcan, Ayhan Koyuncu, Mehmet Fatih Börksüz, Nergiz Hacer Turgut, Fadime Yumuk

**Affiliations:** 1 Cumhuriyet University, School of Medicine, Department of Nuclear Medicine, Sivas, Turkey; 2 Cumhuriyet University, School of Medicine, Department of General Surgery, Sivas, Turkey; 3 Cumhuriyet University, School of Veterinary, Department of Pharmacology and Toxicology, Sivas, Turkey

**Keywords:** I-131, microcarcinoma, differentiated thyroid carcinoma, TNM classification

## Abstract

**Objective:** In this study, we aimed to evaluate the tumor size for proximal and distant metastases when the new and old TNM classification is taken into account in differentiated thyroid cancers.

**Material and Methods:** Two hundred sixty eight patients diagnosed with thyroid carcinoma, undergoing bilateral total or subtotal thyroidectomy treated with high doses of I-131 were examined retrospectively. The data of these patients were compared after classification, according to tumor size <1 cm and <2 cm, lymph node metastases thyroid and tumor capsule invasion at the time of diagnosis, and accumulation of abnormal activity in post I-131 treatment whole-body scan. I-131 uptakes besides physiological and thyroid bed were considered as abnormal activity uptakes.

**Results:** A total of 268 patients with average age of 19-82 yrs (mean: 47.0±13.8 yrs) were included in the study. At postoperative histopathological evaluation, 228 (85.1%) of patients were reported as papillary, 13 (4.9%) as follicular, 23 (8.6%) as well differentiated tumor of unknown malignant potential, 2 (0.7%) as insular and 2 (0.7%) as Hürthle-cell carcinoma. In patients with known tumor size, 96 of 207 (46.4%) patients' tumor size was <1 cm and in 111 (53.6%) >1 cm. In the same group, according to the revised TNM classification, in 149 of 207 patients (72%) the tumor size was <2 cm, whereas in 58 (28%) >2 cm. Of 187 patients with negative lymph nodes, 15 (8%) showed abnormal activity accumulation in the first post I-131 treatment whole-body scan and 10 (40% of 25 patients) positive lymph node (p<0.05) involvement.

**Conclusion:** Since the treatment of patients with microcarcinoma is controversial, tumor size should not be the only factor considered in patients with differentiated thyroid cancer Tissue tumor invasion, age, gender and multifocality should also be taken into account.

**Conflict of interest:**None declared.

## INTRODUCTION

Papillary thyroid carcinoma (PTC), follicular (FTC) and Hurthle cell carcinoma are derived from thyroid follicle cells and termed as differentiated thyroid carcinomas (DTC). PTC incidence is higher than follicular and other types of thyroid cancer and continues to increase with time. The increase of PTC incidence is the main cause of the incidence increase, as 85% of all thyroid cancers (1). It is thought that one of the reasons for this is the increased use of high-tech ultrasound equipment, and the widespread use of fine needle aspiration biopsy (2). Because of the high prevalence com-pared to other thyroid cancers, PTC is the most frequent histological group for mortality. (3). While incidences of FTC and medullary thyroid cancer are relatively stable, there is a reduction in the incidence of anaplastic cancers. Although PTC and FTC derive from thyroid follicular cells, and have different clinical behavior; the former prefers to invade via lymphatics to regional lymph nodes and lung, while the latter to bone by hematogenous way (4). Although lymphatic invasion is frequent in papillary thyroid carcinoma, hematogenous invasion, especially in small lesions is not seen very often. Adjacent lymph node metastases at diagnosis are seen in about the half of the cases (5) while the possibility of seeing distant metastases is much lower. However, the risk of tumor-related mortality is higher for distant metastases compared to local recurrences, due to newly appeared metastases in distant organs from aggressive primary lesions which tend to grow more rapidly (6). Tumor size is an important prognostic factor in PTCs. Cancer-specific mortality rate increases with increasing tumor size (1). The treatment of choice for thyroid cancer is surgery. DTCs capture, organify iodine and have the ability to synthesize and release thyroglobulin (Tg). Because of this feature, DTCs can be treated with high doses of radioactive iodine (I-131) after thyroidectomy. Remnants of thyroid tissue after thyroidectomy are also treated by I-131. Elevated levels of serum Tg (≤2 ng/ml), is a specific indicator with high sensitivity and means presence of residual thyroid tissue, metastatic focus or recurrence (4,7,8). In addition,post-ablation whole-body scintigraphy provides the detection of unknown metastatic foci and treatment of microscopic tumor foci (9). Thus, thyroid carcinomas, even at very small sizes can be determined.

In clinical practice, tumor size ≤1 cm is defined as "microcarcinoma". According to the old tumor, node, and metastasis (TNM) classification, group of patients with tumor size ≤ 1 cm was considered to be T1, whereas in the new TNM classification tumor size ≤2 cm is considered to be T1 (10). Currently, frequently discussed topic is the treatment plan of microcarcinomas. According to the American Thyroid Association (ATA) Manual (10) in patients with a tumor diameter ≤1 cm, low-risk group, one-sided tumor, no history of head and neck radiation, no family history, no clinical and radiological cervical lymph node metastases and patients with the tumor localized in the thyroid, it is suggested that only lobectomy may be sufficient (11,12). However, other authors suggest that in all patient groups total or near total thyroidectomy is recommended. In addiction, some guidelines also recommend total thyroidectomy with neck dissection (13). DTCs are usually curable, with good prognosis, although showing slow progress they can develop recurrence and metastases over time. Apart from tumor size and spread, the patient's age, gender, tumor's histological grade, presence of regional and distant metastases at diagnosis are extremely important factors and have to be taken under serious consideration.

The purpose of this study is to evaluate the relationship between tumor size and lymph node metastasis, capsular invasion and the pathological accumulation in the I-131 whole body scintigraphy by classifying according to the old and new TNM classification.

## MATERIALS AND METHODS

Patients who presented between the years 2007-2010 at Cumhuriyet University Medical School Hospital, diagnosed with thyroid carcinoma, undergoing bilateral total or subtotal thyroidectomy and treated with high doses of radioactive I-131 were examined retrospectively. In this patient group, with lymph node metastasis, thyroid and tumor capsule invasion at the time of diagnosis and in patients who showed accumulation of abnormal activity in whole body scan were compared after classification according to tumor size ≤1 cm and ≤2 cm. The I-131 uptakes besides physiological and thyroid bed were considered as abnormal activity uptakes.

SPSS 15.0 software was used for statistical analysis. The chi-square test was applied to evaluate the statistical significance of the parameters, Significance levels were presented as p values. It was assumed that the observed differences were statically significant at the p ≤ 0.05 levels.

## RESULTS

A total of 268 patients with an age range of 19-82 yrs (mean: 47.0±13.8 yrs) were included in this study. Demographic and clinicopathologic characteristics are listed in Table 1. Patients were given doses of I-131 ranging from 90-314 mCi (mean: 116.4±25.8 mCi) orally. 223 patients (83.2%) were female and 45 (16.8%) were male. Hundred twenty one patients (45.1%) were under the age 45 and 147 patients (54.9%) over 45. At postoperative pathological evaluation 228 (85.1%) of patients were reported as papillary, 13 (4.9%) as follicular, 23 (8.6%) as well differentiated tumor of unknown malignant potential, 2 (0.7%) as insular and 2 (0.7%) as Hurthle cell carcinoma. In 96 of 207 (46.4%) patients tumor size was ≤1 cm and in 111 (53.6%) ≤1 cm. In the same group, in 149 of 207 patients (72%) the tumor size was ≤2 cm and in 58 (28%) ≤2 cm according to the revised TNM classification.

In our study, using both old and new TNM classifications the relationship between tumor size and lymph node metastasis, capsular invasion, and the total body scintigraphy findings after high doses of I-131 was evaluated.

According to lymph node metastases, from 221 patients in whom lymph node involvement was stated, in pathological assessment, 194 (87.8%) lymph node metas- tases were negative, while 27 (12.2%) positive. In 5 (23.8%) of 21 patients with known tumor size and lymph node metastases, tumor size was ≤1 cm and according to the revised TNM there were 8 (38.1%) with tumor size ≤2 cm (Table 2). Of 187 patients with negative lymph nodes in pathological evaluation, 15 (8%) patients showed abnormal activity accumulation on first high-dose radioactive iodine scan, while abnormal accumulation was seen in 10 (40%) of 25 patients with positive lymph nodes (p<0.05). In 34 patients with known tumor size and thyroid capsular invasion, tumor size was ≤1 cm in 8 (23.5%) patients and according to revised TNM classification it was 14 (41.2%) (Table 2). When tumor capsule invasion was noticed, according to the revised TNM classification, of 56 patients with known tumor size and tumor capsular invasion, tumor size was ≤2 cm in 31 (55.4%) patients and according to the old classification, tumor size was ≤1 cm in 17 (30.4%)patients. In posttherapy whole body scans in patients who received high doses of I-31 after surgical excision, 5 (21.7%) were positive with tumor size ≤1 cm and 10 (43.5%) with tumor size ≤2 cm (Table 2).

## DISCUSSION

Thyroid carcinomas are the most common cancers of the endocrine system and papillary carcinoma is the most widespread type. In a study done by Davies et al (14) which evaluates the patient groups between the years 1973-2002 an increased incidence of papillary cancer was seen and no significant change in the incidence of follicular, medullary and anaplastic cancers was observed. It is thought that the increase in the incidence of papillary cancer depends on the developments in ultrasonography and fine-needle aspiration biopsy and this also leads to increase in the detection of small size cancers. In the same study, for the group with tumor size ≤1 cm, a 49% incidence increase was found while for those ≤2 cm an 87% increase was observed. It was shown that despite the increase in incidence, mortality remained stable (14). In our study, 228 (85.1%) of 268 patients had papillary cancer.

The prognosis of thyroid cancer is excellent when appropriate treatment is applied. Ten year survival is reported in an approximately 85% of the cohort and in those with distant metastasis a 25-40% was referred (15). The presence of many factors such as age, sex, tumor histological grade, tumor size, multifocality, regional and distant metastases at diagnosis is effective on prognosis of differentiated thyroid carcinomas. The large size of the tumor, the presence of lymph node metastases, spread beyond the thyroid capsule, advanced age and male gender have been considered as bad prognostic factors (16). Papillary carcinomas prefer to invade regional lymph nodes and lung, while follicular carcinomas, go to the bone by hematogenous way (4). Approximately half of the patients with PTC have lymph node metastases at diagnosis (5). The effects of the presence of lymph node metastases on thyroid cancer prognosis are known. Especially the presence of bilateral or cervical lymph node metastases or invasion at lymph node capsule are bad prognostic features increasing the risk of regional and distant metastases (7,17). Machens et al. (18) found the probability of lymphogenic micrometastasis significantly increased in papillary thyroid cancer above a tumor diameter cut-off of 5 mm. Therefore, the small PTC is not always synonymous with a low-risk constellation, especially considering that lymph node spread is a risk factor for distant metastasis in PTC. In clinically lymph node-negative patients, usually during surgery or during the pathological examination of the material removed, lymph node metastases can be detected. In most patients lymph node metastases develop at the central compartment (10). In one study, in patients with clinically lymph node-negative papillary thyroid microcarcinoma, subclinical central lymph node metastases have been reported in a 36.7%. However, it has been also stated that the removal of undetected central lymph nodes prior to the surgery as prophylactic technique has low benefit in the prognosis (19). In our patient group, abnormal accumulation of activity was seen on the first high-dose I-131 scintigraphy in 15 of 187 (8%) patients with negative lymph nodes; on screening scan abnormal activity was noticed in 10 of 25 (40%) patients with positive lymph node involvement.

The actual cause of death in PTC is the distant metastases. According to a study done by Ozkan et al (20), worse prognosis is found in patients who developed distant metastasis during follow-up compared to those with distant metastases at first diagnosis. Differentiated thyroid carcinomas have the ability to capture and organify iodine and also synthesize and release thyroglobulin. Because of this feature, DTCs can be treated with high doses of 131I after thyroidectomy. Complete removal of the thyroid is rare and in most cases with post-operative I-131 scintigraphy, functioning thyroid tissue is detected (21). Thyroidectomy is thus completed with total ablation. Whole body scintigraphy taken after treatment with I-131 is very valuable in predicting prognosis and deciding if additional treatment may be needed. The sensitivity for residual tumor and recurrence is 50-90%, specificity is 80-100% (22). While there is 10-year survival in 92% of patients with negative I-131 scintigraphy at treatment dose, this rate falls to 19% in those with positive scan (1). In our study, in 40% of patients with positive lymph nodes, pathological activity accumulation was observed in the post I-131 treatment whole-body scan. In addition, we observed pathological uptake outside the thyroid bed in 5 of 90 (5.6%) patients with tumor size ≤1 cm in the whole body scan after I-131 treatment. In the whole body scan of the same group of patients with regard to the new TNM classification, pathological uptake was seen in 10 of 141 (7.1%) patients.

Mazzaferri et al. (17) have reported that recurrence significantly decreases in patients with papillary cancer who had total or subtotal thyroidectomy, ablation with I-131 and L-thyroxine therapy compared to patients who had only total thyroidectomy and treated with L-thyroxine. Recurrence or residue in DTCs is important indicators of morbidity and these are troubled conditions requiring new therapeutic applications (3). In a study in the follow-up of patients who received total thyroidectomy and successful ablation, no significant difference was found between high risk group and low risk group in terms of recurrence (23). 

In a study done by Ito et al (24), in 2638 patients with tumor diameter ≤2 cm and being T1N0M0 without radioactive iodine treatment a 0.2% developed distant metastases, and one patient died of thyroid cancer. In another study, in which Fukushima et al (25) evaluated 5917 patients, 2 patients developed distant metastases with tumor size ≤1 (n=1,261), whereas in 40 local recurrence was detected. In patients with tumor size 1.1-2 cm, 16 distant metastases were depicted and 85 local recurrences were detected. Of these patients, 2 with tumor size ≤1 cm and 8 with tumor size 1.1-2 cm died. Only one of our patients died 5.4 years after diagnosis due to brain metastasis of thyroid carcinoma. And this patient's tumor size at diagnosis was reported as 7 mm.

The differentiated thyroid cancer follow-up protocol should be determined on the basis of the first treatment results instead of the classification of the tumor at first diagnosis. To make a risk classification according to ATA's guide, the risk should be determined for patients after receiving treatment with I-131 following total thyroidectomy and the follow-up protocol should be produced according to this evaluation.

## CONCLUSION

We think that at the approach to thyroid cancer, particularly papillary thyroid cancer, instead of determining the treatment plan considering the tumor size either ≤1 cm, or ≤2 cm in the first place; the invasion of the tumor (lymph node, thyroid capsule, the surrounding tissue, etc.), patient age, gender, and multifocality in the surgical and clinical approach would be more accurate. Determining the treatment plan tumor size should not be the only factor of choice in papillary thyroid cancer. A plethora of other factors has to be taken into account like tissue tumor invasion, age, gender and multifocality.

## Figures and Tables

**Table 1:**
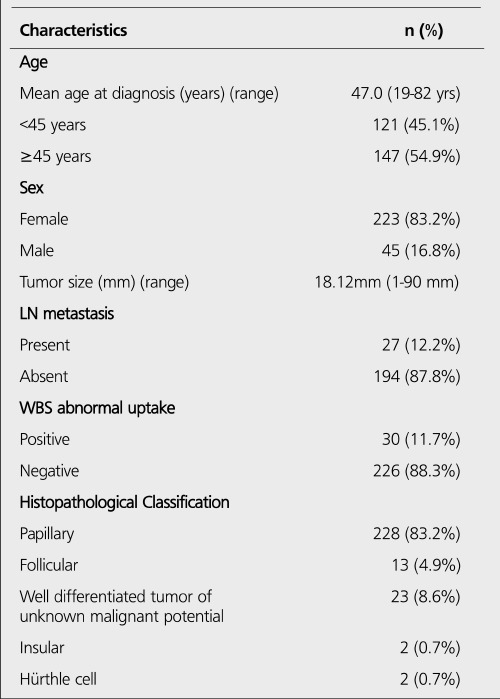
Demographic and clinicopathologic characteristics

**Table 2:**
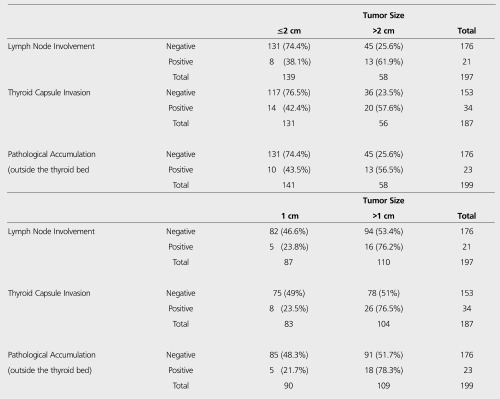
The relationship between the tumor size and lymph node metastasis, thyroid capsular invasion, and the post-131I treatment whole-bodyscan findings
